# 2,2′-[1,1′-(Propane-1,3-diyldioxy­dinitrilo)diethyl­idyne]di-1-naphthol

**DOI:** 10.1107/S1600536809023241

**Published:** 2009-06-20

**Authors:** Wen-Kui Dong, Jian-Chao Wu, Yin-Xia Sun, Li Li, Jian Yao

**Affiliations:** a School of Chemical and Biological Engineering, Lanzhou Jiaotong University, Lanzhou 730070, People’s Republic of China

## Abstract

The mol­ecule of the title compound, C_27_H_26_N_2_O_4_, lies across a crystallographic inversion centre and adopts an l-shaped configuration. Within the mol­ecule, the two naphthalene units are approximately perpendicular, making a dihedral angle of 80.24 (5)°. The two intramolecular O—H⋯N hydrogen bonds, generate *S*(6) ring motifs. In the crystal structure, every mol­ecule links five other mol­ecules into an infinite cross-linked layered supra­molecular structure *via* inter­molecular C—H⋯O hydrogen bonds, C—H⋯π inter­actions and π–π stacking inter­actions [centroid–centroid distance = 3.956 (4) Å].

## Related literature

For the steric and electronic properties of Schiff bases, see: Yamada (1999 ▶). For background to this study, see: Dong *et al.* (2006 ▶). For related structures, see: Dong & Duan (2008 ▶); Dong *et al.* (2008*a* ▶,*b* ▶,*c* ▶,*d* ▶); Duan *et al.* (2007 ▶); He *et al.* (2008 ▶).
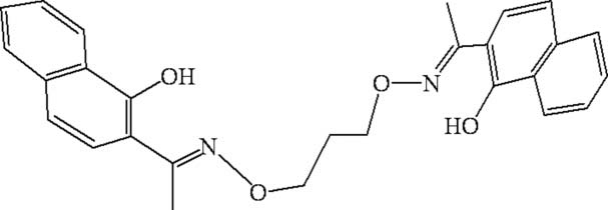


## Experimental

### 

#### Crystal data

C_27_H_26_N_2_O_4_
*M* *_r_* = 442.50
Triclinic, 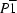

*a* = 7.4411 (10) Å
*b* = 8.8911 (16) Å
*c* = 18.106 (2) Å
α = 100.645 (1)°
β = 94.331 (1)°
γ = 106.329 (2)°
*V* = 1119.4 (3) Å^3^
*Z* = 2
Mo *K*α radiation
μ = 0.09 mm^−1^
*T* = 298 K
0.50 × 0.42 × 0.37 mm

#### Data collection

Siemens SMART 1000 CCD area-detector diffractometer
Absorption correction: multi-scan (*SADABS*; Sheldrick, 1996 ▶) *T*_min_ = 0.957, *T*_max_ = 0.968
5800 measured reflections
3876 independent reflections
2325 reflections with *I* > 2σ(*I*)
*R*_int_ = 0.025

#### Refinement

*R*[*F* ^2^ > 2σ(*F* ^2^)] = 0.047
*wR*(*F* ^2^) = 0.145
*S* = 1.04
3876 reflections
298 parameters
H-atom parameters constrained
Δρ_max_ = 0.22 e Å^−3^
Δρ_min_ = −0.22 e Å^−3^

### 

Data collection: *SMART* (Siemens, 1996 ▶); cell refinement: *SAINT* (Siemens, 1996 ▶); data reduction: *SAINT*; program(s) used to solve structure: *SHELXS97* (Sheldrick, 2008 ▶); program(s) used to refine structure: *SHELXL97* (Sheldrick, 2008 ▶); molecular graphics: *SHELXTL* (Sheldrick, 2008 ▶); software used to prepare material for publication: *SHELXTL*.

## Supplementary Material

Crystal structure: contains datablocks global, I. DOI: 10.1107/S1600536809023241/hg2527sup1.cif

Structure factors: contains datablocks I. DOI: 10.1107/S1600536809023241/hg2527Isup2.hkl

Additional supplementary materials:  crystallographic information; 3D view; checkCIF report

## Figures and Tables

**Table 1:** Hydrogen-bond geometry (Å, °)

*D*—H⋯*A*	*D*—H	H⋯*A*	*D*⋯*A*	*D*—H⋯*A*
O3—H3⋯N1	0.82	1.83	2.543 (2)	145
O4—H4⋯N2	0.82	1.82	2.540 (2)	146
C12—H12⋯O3^i^	0.93	2.68	3.588 (3)	166
C1—H1*B*⋯*Cg*1^ii^	0.97	2.78	3.480 (2)	129

Symmetry codes: (i) 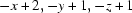; (ii) 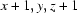. *Cg*1 is the centroid of the C6–C15 ring.

**Fig. 1.**
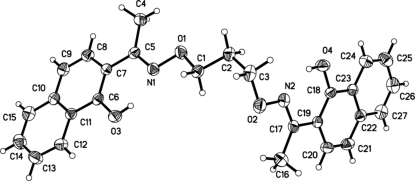
The molecular structure of the title compound with atom numbering scheme. Displacement ellipsoids for non-hydrogen atoms are drawn at the 30% probability level.

**Fig. 2.**
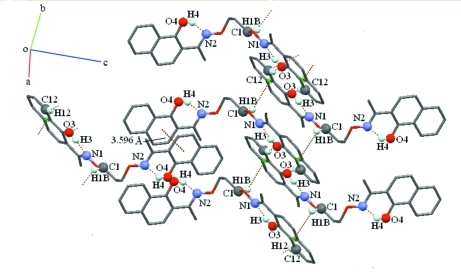
Part of the supramolecular structure of the title compound. intra- and intermolecular hydrogen bonds, C—H···π interaction and π–π stacking interactions are shown as dashed lines.

## supplementary crystallographic information

### Comment

Schiff bases are compounds containing the azomethine group, –*R*,*R*'C=N, prepared by the condensation reaction of a primary amine with active carbonyl group. Due to the versatility of their steric and electronic properties (Yamada, 1999), which can be fine tuned by choosing the appropriate amine and the substituents on an aromatic ring of the carbonyl compound, Schiff base bisoxime compounds have gained increased interest in the field of coordination chemistry (Dong *et al.*, 2008*a*; He *et al.*, 2008). As a part of our ongoing research (Dong *et al.*, 2006; Duan *et al.*, 2007), the synthesis and crystal structure of the title compound was reported (Fig. 1).

The molecule of the title compound lies across a crystallographic inversion centre (symmetry code: -*x*, -*y*, -*z*) and adopts an L-shaped configuration. This structure is not similar to what was observed in our previously reported series oxime compounds containing four-methene bridge, which always adopt a V-shaped configurations (Dong *et al.*, 2006; Duan *et al.*, 2007; Dong *et al.*, 2008*a*; Dong & Duan, 2008; Dong *et al.*, 2008*b*; Dong *et al.*, 2008d; Dong *et al.*, 2008*c*; He *et al.*, 2008). Within the molecule, the dihedral angle between the plane of oxime functional groups and naphthalene ring is 8.93 (3)° for C6—C15 ring and O1—N1—C5, 5.30 (3)° for C18—C27 ring and O2—N2—C17, respectively. And the two naphthalene units are approximately vertical with the dihedral angle of 80.24 (5)°. The two intramolecular hydrogen bonds, O3—H3···N1 and O4—H4···N2,generate S(6) ring motifs helping to the stabilization of the title molecule.

In the crystal structure, the crystals are held together by an intermolecular C—H···π interaction and C12—H12···O3 hydrogen bonds between the phenolic-oxygen atom and the hydrogen of the naphthalene ring, in which the C1—H1B···π centroid separations are equal 2.782 Å involving the naphthalene ring C6—C15 (centroid, *Cg*1). In addition, the adjacent aromatic rings are further linked by the intermolecular π–π stacking interactions [centroid-to-centroid distance = 3.596 (4) Å]. Thus, every title compound molecule links five other molecules into an infinite crosslinked layer supramolecular structure *via* intermolecular C—H···O hydrogen bonds, C—H···π and π–π stacking interactions (Fig. 2).

### Experimental

2,2'-[(Propane-1,3-diyldioxy)bis(nitriloethylidyne)]dinaphthol was synthesized according to an analogous method reported earlier (Dong *et al.*, 2008 e). To an ethanol solution (5 ml) of 2-acetyl-1-naphthol (388.5 mg, 2.06 mmol) was added dropwise an ethanol solution (3 ml) of 1,3-bis(aminooxy)propane (109.7 mg, 1.03 mmol). The mixture solution was stirred at 328–333 K for 72 h. After cooling to room temperature, the precipitate was filtered off, and washed successively three times with ethanol. The product was dried *in vacuo* and purified by recrystallization from ethanol to yield 320.4 mg (Yield, 70.0%) of powder; m.p. 439–441 K. Colorless block-like single crystals suitable for X-ray diffraction studies were obtained by slow evaporation from a solution of ethyl acetate of 2,2'-[(propane-1,3-diyldioxy)bis(nitriloethylidyne)]dinaphthol at room temperature for about one month. Anal. Calcd. for C_27_H_26_N_2_O_4_: C, 73.28; H, 5.92; N, 6.33; Found: C, 73.25; H, 5.97; N, 6.29.

### Refinement

Non-H atoms were refined anisotropically. H atoms were treated as riding atoms with distances C—H = 0.97 (CH_2_), C—H = 0.96 (CH_3_), 0.93 Å (CH), 0.82 Å (OH), and *U*_iso_(H) = 1.2 *U*_eq_(C) and 1.5 *U*_eq_(O).

### Crystal data

**Table:**

C_27_H_26_N_2_O_4_	*Z* = 2
*M**_r_* = 442.50	*F*(000) = 468
Triclinic, *P*1	*D*_x_ = 1.313 Mg m^−^^3^
Hall symbol: -P 1	Mo *K*α radiation, λ = 0.71073 Å
*a* = 7.4411 (10) Å	Cell parameters from 1962 reflections
*b* = 8.8911 (16) Å	θ = 2.3–26.3°
*c* = 18.106 (2) Å	µ = 0.09 mm^−^^1^
α = 100.645 (1)°	*T* = 298 K
β = 94.331 (1)°	Block-like, colorless
γ = 106.329 (2)°	0.50 × 0.42 × 0.37 mm
*V* = 1119.4 (3) Å^3^	

### Data collection

**Table:**

Siemens SMART 1000 CCD area-detector diffractometer	3876 independent reflections
Radiation source: fine-focus sealed tube	2325 reflections with *I* > 2σ(*I*)
graphite	*R*_int_ = 0.025
φ and ω scans	θ_max_ = 25.0°, θ_min_ = 2.3°
Absorption correction: multi-scan (*SADABS*; Sheldrick, 1996)	*h* = −6→8
*T*_min_ = 0.957, *T*_max_ = 0.968	*k* = −10→10
5800 measured reflections	*l* = −20→21

### Refinement

**Table:**

Refinement on *F*^2^	Primary atom site location: structure-invariant direct methods
Least-squares matrix: full	Secondary atom site location: difference Fourier map
*R*[*F*^2^ > 2σ(*F*^2^)] = 0.047	Hydrogen site location: inferred from neighbouring sites
*wR*(*F*^2^) = 0.145	H-atom parameters constrained
*S* = 1.04	*w* = 1/[σ^2^(*F*_o_^2^) + (0.0669*P*)^2^ + 0.1652*P*] where *P* = (*F*_o_^2^ + 2*F*_c_^2^)/3
3876 reflections	(Δ/σ)_max_ < 0.001
298 parameters	Δρ_max_ = 0.22 e Å^−^^3^
0 restraints	Δρ_min_ = −0.22 e Å^−^^3^

### Special details

**Table:**

Geometry. All e.s.d.'s (except the e.s.d. in the dihedral angle between two l.s. planes) are estimated using the full covariance matrix. The cell e.s.d.'s are taken into account individually in the estimation of e.s.d.'s in distances, angles and torsion angles; correlations between e.s.d.'s in cell parameters are only used when they are defined by crystal symmetry. An approximate (isotropic) treatment of cell e.s.d.'s is used for estimating e.s.d.'s involving l.s. planes.
Refinement. Refinement of *F*^2^ against ALL reflections. The weighted *R*-factor *wR* and goodness of fit *S* are based on *F*^2^, conventional *R*-factors *R* are based on *F*, with *F* set to zero for negative *F*^2^. The threshold expression of *F*^2^ > σ(*F*^2^) is used only for calculating *R*-factors(gt) *etc*. and is not relevant to the choice of reflections for refinement. *R*-factors based on *F*^2^ are statistically about twice as large as those based on *F*, and *R*- factors based on ALL data will be even larger.

### Fractional atomic coordinates and isotropic or equivalent isotropic displacement parameters (Å^2^)

**Table:**

	*x*	*y*	*z*	*U*_iso_*/*U*_eq_	
N1	0.4304 (3)	0.3291 (2)	0.39356 (10)	0.0472 (5)	
N2	0.5352 (3)	0.4359 (2)	0.09736 (10)	0.0490 (5)	
O1	0.3227 (2)	0.35088 (19)	0.33130 (9)	0.0531 (5)	
O2	0.6102 (2)	0.55995 (19)	0.16190 (9)	0.0568 (5)	
O3	0.7381 (2)	0.38979 (19)	0.48069 (9)	0.0567 (5)	
H3	0.6693	0.3990	0.4453	0.085*	
O4	0.2690 (2)	0.2606 (2)	−0.00541 (9)	0.0589 (5)	
H4	0.3176	0.3292	0.0332	0.088*	
C1	0.4460 (3)	0.4646 (3)	0.29743 (12)	0.0444 (6)	
H1A	0.5504	0.4264	0.2822	0.053*	
H1B	0.4971	0.5663	0.3336	0.053*	
C2	0.3361 (3)	0.4864 (3)	0.22958 (12)	0.0494 (6)	
H2A	0.2300	0.5216	0.2451	0.059*	
H2B	0.2864	0.3843	0.1936	0.059*	
C3	0.4578 (4)	0.6083 (3)	0.19140 (13)	0.0557 (7)	
H3A	0.3795	0.6253	0.1503	0.067*	
H3B	0.5088	0.7097	0.2278	0.067*	
C4	0.1200 (3)	0.1930 (3)	0.42415 (14)	0.0608 (7)	
H4A	0.0746	0.2444	0.3878	0.091*	
H4B	0.0718	0.2184	0.4710	0.091*	
H4C	0.0779	0.0789	0.4052	0.091*	
C5	0.3322 (3)	0.2511 (2)	0.43736 (12)	0.0421 (5)	
C6	0.6336 (3)	0.2932 (2)	0.52118 (12)	0.0409 (5)	
C7	0.4391 (3)	0.2236 (2)	0.50250 (11)	0.0385 (5)	
C8	0.3456 (3)	0.1201 (3)	0.54803 (13)	0.0488 (6)	
H8	0.2156	0.0725	0.5366	0.059*	
C9	0.4393 (4)	0.0885 (3)	0.60760 (13)	0.0510 (6)	
H9	0.3730	0.0189	0.6356	0.061*	
C10	0.6365 (3)	0.1598 (3)	0.62776 (12)	0.0445 (6)	
C11	0.7339 (3)	0.2654 (2)	0.58449 (12)	0.0413 (5)	
C12	0.9311 (3)	0.3417 (3)	0.60585 (14)	0.0552 (7)	
H12	0.9972	0.4118	0.5781	0.066*	
C13	1.0230 (4)	0.3126 (3)	0.66666 (16)	0.0673 (8)	
H13	1.1520	0.3636	0.6803	0.081*	
C14	0.9279 (4)	0.2076 (3)	0.70901 (16)	0.0667 (8)	
H14	0.9933	0.1888	0.7504	0.080*	
C15	0.7397 (4)	0.1326 (3)	0.69001 (14)	0.0585 (7)	
H15	0.6777	0.0621	0.7185	0.070*	
C16	0.8658 (4)	0.4430 (3)	0.09754 (15)	0.0681 (8)	
H16A	0.8829	0.5187	0.1447	0.102*	
H16B	0.9350	0.4952	0.0620	0.102*	
H16C	0.9112	0.3557	0.1058	0.102*	
C17	0.6600 (3)	0.3798 (3)	0.06659 (12)	0.0449 (6)	
C18	0.4008 (3)	0.1992 (3)	−0.03454 (12)	0.0419 (5)	
C19	0.5894 (3)	0.2506 (3)	−0.00192 (12)	0.0419 (5)	
C20	0.7126 (3)	0.1727 (3)	−0.03704 (14)	0.0518 (6)	
H20	0.8390	0.2047	−0.0161	0.062*	
C21	0.6529 (3)	0.0533 (3)	−0.10000 (14)	0.0552 (6)	
H21	0.7382	0.0044	−0.1209	0.066*	
C22	0.4635 (3)	0.0022 (3)	−0.13423 (13)	0.0460 (6)	
C23	0.3358 (3)	0.0756 (3)	−0.10137 (12)	0.0422 (5)	
C24	0.1464 (4)	0.0254 (3)	−0.13600 (14)	0.0582 (7)	
H24	0.0612	0.0739	−0.1147	0.070*	
C25	0.0862 (4)	−0.0921 (3)	−0.19974 (16)	0.0677 (8)	
H25	−0.0393	−0.1237	−0.2217	0.081*	
C26	0.2131 (4)	−0.1659 (3)	−0.23247 (15)	0.0669 (8)	
H26	0.1715	−0.2467	−0.2761	0.080*	
C27	0.3962 (4)	−0.1200 (3)	−0.20072 (14)	0.0604 (7)	
H27	0.4792	−0.1697	−0.2231	0.072*	

### Atomic displacement parameters (Å^2^)

**Table:**

	*U*^11^	*U*^22^	*U*^33^	*U*^12^	*U*^13^	*U*^23^
N1	0.0453 (12)	0.0502 (11)	0.0435 (11)	0.0087 (9)	0.0039 (9)	0.0134 (9)
N2	0.0551 (13)	0.0511 (12)	0.0396 (11)	0.0148 (10)	0.0066 (10)	0.0086 (9)
O1	0.0478 (10)	0.0605 (10)	0.0468 (9)	0.0058 (8)	0.0015 (8)	0.0198 (8)
O2	0.0626 (11)	0.0590 (11)	0.0434 (10)	0.0119 (9)	0.0087 (8)	0.0069 (8)
O3	0.0413 (9)	0.0685 (11)	0.0573 (10)	0.0017 (8)	0.0116 (8)	0.0277 (9)
O4	0.0453 (10)	0.0706 (12)	0.0605 (11)	0.0237 (9)	0.0085 (8)	0.0031 (9)
C1	0.0471 (14)	0.0396 (12)	0.0447 (13)	0.0090 (11)	0.0113 (11)	0.0087 (10)
C2	0.0585 (16)	0.0495 (14)	0.0412 (13)	0.0188 (12)	0.0078 (11)	0.0081 (11)
C3	0.0747 (19)	0.0512 (15)	0.0442 (14)	0.0223 (13)	0.0116 (13)	0.0113 (11)
C4	0.0441 (15)	0.0763 (18)	0.0544 (16)	0.0052 (13)	0.0058 (12)	0.0158 (14)
C5	0.0420 (13)	0.0375 (12)	0.0426 (13)	0.0075 (10)	0.0103 (11)	0.0034 (10)
C6	0.0445 (13)	0.0351 (12)	0.0408 (13)	0.0067 (10)	0.0145 (11)	0.0071 (10)
C7	0.0403 (13)	0.0343 (11)	0.0376 (12)	0.0070 (10)	0.0084 (10)	0.0053 (9)
C8	0.0430 (13)	0.0431 (13)	0.0539 (15)	0.0020 (11)	0.0112 (12)	0.0102 (11)
C9	0.0600 (16)	0.0413 (13)	0.0504 (15)	0.0067 (12)	0.0157 (13)	0.0164 (11)
C10	0.0529 (15)	0.0363 (12)	0.0448 (14)	0.0150 (11)	0.0103 (12)	0.0054 (10)
C11	0.0424 (13)	0.0393 (12)	0.0417 (13)	0.0127 (10)	0.0088 (10)	0.0052 (10)
C12	0.0428 (14)	0.0619 (16)	0.0589 (16)	0.0134 (12)	0.0089 (12)	0.0107 (13)
C13	0.0509 (16)	0.0782 (19)	0.0696 (19)	0.0214 (14)	−0.0028 (14)	0.0092 (16)
C14	0.076 (2)	0.0709 (18)	0.0596 (17)	0.0337 (16)	−0.0010 (15)	0.0152 (14)
C15	0.0749 (19)	0.0511 (15)	0.0553 (16)	0.0245 (14)	0.0129 (14)	0.0161 (12)
C16	0.0544 (17)	0.083 (2)	0.0582 (17)	0.0127 (14)	0.0003 (13)	0.0089 (14)
C17	0.0450 (14)	0.0517 (14)	0.0406 (13)	0.0115 (11)	0.0052 (11)	0.0213 (11)
C18	0.0390 (13)	0.0483 (13)	0.0447 (13)	0.0163 (11)	0.0123 (11)	0.0180 (11)
C19	0.0415 (13)	0.0496 (13)	0.0389 (13)	0.0142 (11)	0.0081 (10)	0.0187 (10)
C20	0.0400 (14)	0.0594 (16)	0.0589 (16)	0.0165 (12)	0.0073 (12)	0.0170 (13)
C21	0.0497 (16)	0.0591 (16)	0.0639 (17)	0.0251 (13)	0.0170 (13)	0.0137 (13)
C22	0.0502 (15)	0.0424 (13)	0.0500 (14)	0.0145 (11)	0.0118 (12)	0.0182 (11)
C23	0.0428 (13)	0.0426 (13)	0.0447 (13)	0.0129 (10)	0.0072 (11)	0.0175 (10)
C24	0.0511 (16)	0.0580 (16)	0.0629 (17)	0.0149 (13)	0.0031 (13)	0.0110 (13)
C25	0.0582 (17)	0.0597 (17)	0.074 (2)	0.0070 (14)	−0.0095 (15)	0.0113 (15)
C26	0.083 (2)	0.0475 (15)	0.0576 (17)	0.0083 (15)	−0.0031 (16)	0.0039 (13)
C27	0.074 (2)	0.0469 (15)	0.0600 (17)	0.0194 (14)	0.0116 (15)	0.0090 (12)

### Geometric parameters (Å, °)

**Table:**

N1—C5	1.284 (3)	C10—C11	1.413 (3)
N1—O1	1.408 (2)	C11—C12	1.422 (3)
N2—C17	1.287 (3)	C12—C13	1.356 (3)
N2—O2	1.404 (2)	C12—H12	0.9300
O1—C1	1.426 (2)	C13—C14	1.391 (4)
O2—C3	1.427 (3)	C13—H13	0.9300
O3—C6	1.350 (2)	C14—C15	1.357 (4)
O3—H3	0.8200	C14—H14	0.9300
O4—C18	1.345 (3)	C15—H15	0.9300
O4—H4	0.8200	C16—C17	1.498 (3)
C1—C2	1.497 (3)	C16—H16A	0.9600
C1—H1A	0.9700	C16—H16B	0.9600
C1—H1B	0.9700	C16—H16C	0.9600
C2—C3	1.514 (3)	C17—C19	1.473 (3)
C2—H2A	0.9700	C18—C19	1.393 (3)
C2—H2B	0.9700	C18—C23	1.424 (3)
C3—H3A	0.9700	C19—C20	1.417 (3)
C3—H3B	0.9700	C20—C21	1.355 (3)
C4—C5	1.502 (3)	C20—H20	0.9300
C4—H4A	0.9600	C21—C22	1.408 (3)
C4—H4B	0.9600	C21—H21	0.9300
C4—H4C	0.9600	C22—C23	1.405 (3)
C5—C7	1.469 (3)	C22—C27	1.414 (3)
C6—C7	1.392 (3)	C23—C24	1.410 (3)
C6—C11	1.418 (3)	C24—C25	1.356 (3)
C7—C8	1.420 (3)	C24—H24	0.9300
C8—C9	1.356 (3)	C25—C26	1.401 (4)
C8—H8	0.9300	C25—H25	0.9300
C9—C10	1.414 (3)	C26—C27	1.355 (4)
C9—H9	0.9300	C26—H26	0.9300
C10—C15	1.412 (3)	C27—H27	0.9300
			
C5—N1—O1	114.40 (18)	C13—C12—C11	120.2 (2)
C17—N2—O2	113.84 (19)	C13—C12—H12	119.9
N1—O1—C1	107.51 (15)	C11—C12—H12	119.9
N2—O2—C3	108.21 (17)	C12—C13—C14	121.1 (3)
C6—O3—H3	109.5	C12—C13—H13	119.4
C18—O4—H4	109.5	C14—C13—H13	119.4
O1—C1—C2	108.60 (18)	C15—C14—C13	120.1 (3)
O1—C1—H1A	110.0	C15—C14—H14	119.9
C2—C1—H1A	110.0	C13—C14—H14	119.9
O1—C1—H1B	110.0	C14—C15—C10	121.1 (2)
C2—C1—H1B	110.0	C14—C15—H15	119.4
H1A—C1—H1B	108.4	C10—C15—H15	119.4
C1—C2—C3	111.5 (2)	C17—C16—H16A	109.5
C1—C2—H2A	109.3	C17—C16—H16B	109.5
C3—C2—H2A	109.3	H16A—C16—H16B	109.5
C1—C2—H2B	109.3	C17—C16—H16C	109.5
C3—C2—H2B	109.3	H16A—C16—H16C	109.5
H2A—C2—H2B	108.0	H16B—C16—H16C	109.5
O2—C3—C2	112.91 (19)	N2—C17—C19	116.1 (2)
O2—C3—H3A	109.0	N2—C17—C16	122.3 (2)
C2—C3—H3A	109.0	C19—C17—C16	121.5 (2)
O2—C3—H3B	109.0	O4—C18—C19	123.0 (2)
C2—C3—H3B	109.0	O4—C18—C23	115.8 (2)
H3A—C3—H3B	107.8	C19—C18—C23	121.2 (2)
C5—C4—H4A	109.5	C18—C19—C20	117.3 (2)
C5—C4—H4B	109.5	C18—C19—C17	122.1 (2)
H4A—C4—H4B	109.5	C20—C19—C17	120.6 (2)
C5—C4—H4C	109.5	C21—C20—C19	122.4 (2)
H4A—C4—H4C	109.5	C21—C20—H20	118.8
H4B—C4—H4C	109.5	C19—C20—H20	118.8
N1—C5—C7	116.2 (2)	C20—C21—C22	120.9 (2)
N1—C5—C4	122.4 (2)	C20—C21—H21	119.6
C7—C5—C4	121.34 (19)	C22—C21—H21	119.6
O3—C6—C7	122.7 (2)	C23—C22—C21	118.9 (2)
O3—C6—C11	116.03 (19)	C23—C22—C27	118.5 (2)
C7—C6—C11	121.28 (19)	C21—C22—C27	122.6 (2)
C6—C7—C8	117.5 (2)	C22—C23—C24	118.9 (2)
C6—C7—C5	122.24 (19)	C22—C23—C18	119.4 (2)
C8—C7—C5	120.3 (2)	C24—C23—C18	121.7 (2)
C9—C8—C7	122.2 (2)	C25—C24—C23	121.2 (3)
C9—C8—H8	118.9	C25—C24—H24	119.4
C7—C8—H8	118.9	C23—C24—H24	119.4
C8—C9—C10	120.9 (2)	C24—C25—C26	120.0 (3)
C8—C9—H9	119.5	C24—C25—H25	120.0
C10—C9—H9	119.5	C26—C25—H25	120.0
C15—C10—C11	118.6 (2)	C27—C26—C25	120.2 (2)
C15—C10—C9	123.0 (2)	C27—C26—H26	119.9
C11—C10—C9	118.4 (2)	C25—C26—H26	119.9
C10—C11—C6	119.6 (2)	C26—C27—C22	121.2 (3)
C10—C11—C12	118.8 (2)	C26—C27—H27	119.4
C6—C11—C12	121.6 (2)	C22—C27—H27	119.4
			
C5—N1—O1—C1	168.65 (19)	C13—C14—C15—C10	−0.5 (4)
C17—N2—O2—C3	−178.59 (18)	C11—C10—C15—C14	1.0 (4)
N1—O1—C1—C2	178.37 (17)	C9—C10—C15—C14	−177.7 (2)
O1—C1—C2—C3	178.99 (18)	O2—N2—C17—C19	−179.28 (16)
N2—O2—C3—C2	72.6 (2)	O2—N2—C17—C16	−0.2 (3)
C1—C2—C3—O2	63.2 (3)	O4—C18—C19—C20	178.18 (19)
O1—N1—C5—C7	179.14 (16)	C23—C18—C19—C20	−1.3 (3)
O1—N1—C5—C4	−1.8 (3)	O4—C18—C19—C17	−1.4 (3)
O3—C6—C7—C8	178.1 (2)	C23—C18—C19—C17	179.14 (19)
C11—C6—C7—C8	−1.7 (3)	N2—C17—C19—C18	4.1 (3)
O3—C6—C7—C5	−0.5 (3)	C16—C17—C19—C18	−175.1 (2)
C11—C6—C7—C5	179.8 (2)	N2—C17—C19—C20	−175.5 (2)
N1—C5—C7—C6	7.2 (3)	C16—C17—C19—C20	5.4 (3)
C4—C5—C7—C6	−171.8 (2)	C18—C19—C20—C21	0.3 (3)
N1—C5—C7—C8	−171.3 (2)	C17—C19—C20—C21	179.9 (2)
C4—C5—C7—C8	9.7 (3)	C19—C20—C21—C22	0.8 (4)
C6—C7—C8—C9	−0.1 (3)	C20—C21—C22—C23	−0.9 (3)
C5—C7—C8—C9	178.5 (2)	C20—C21—C22—C27	178.8 (2)
C7—C8—C9—C10	0.8 (4)	C21—C22—C23—C24	179.4 (2)
C8—C9—C10—C15	178.9 (2)	C27—C22—C23—C24	−0.3 (3)
C8—C9—C10—C11	0.2 (3)	C21—C22—C23—C18	−0.1 (3)
C15—C10—C11—C6	179.3 (2)	C27—C22—C23—C18	−179.8 (2)
C9—C10—C11—C6	−1.9 (3)	O4—C18—C23—C22	−178.33 (19)
C15—C10—C11—C12	−0.8 (3)	C19—C18—C23—C22	1.2 (3)
C9—C10—C11—C12	178.0 (2)	O4—C18—C23—C24	2.2 (3)
O3—C6—C11—C10	−177.05 (19)	C19—C18—C23—C24	−178.3 (2)
C7—C6—C11—C10	2.7 (3)	C22—C23—C24—C25	0.3 (3)
O3—C6—C11—C12	3.1 (3)	C18—C23—C24—C25	179.8 (2)
C7—C6—C11—C12	−177.2 (2)	C23—C24—C25—C26	−0.1 (4)
C10—C11—C12—C13	0.2 (3)	C24—C25—C26—C27	−0.1 (4)
C6—C11—C12—C13	−179.9 (2)	C25—C26—C27—C22	0.2 (4)
C11—C12—C13—C14	0.3 (4)	C23—C22—C27—C26	0.1 (3)
C12—C13—C14—C15	−0.2 (4)	C21—C22—C27—C26	−179.7 (2)

### Hydrogen-bond geometry (Å, °)

**Table:**

*D*—H···*A*	*D*—H	H···*A*	*D*···*A*	*D*—H···*A*
O3—H3···N1	0.82	1.83	2.543 (2)	145
O4—H4···N2	0.82	1.82	2.540 (2)	146
C12—H12···O3^i^	0.93	2.68	3.588 (3)	166
C1—H1B···Cg1^ii^	0.97	2.78	3.480 (2)	129

Symmetry codes: (i) −*x*+2, −*y*+1, −*z*+1; (ii) *x*+1, *y*, *z*+1.
